# Supplementary material for: The kinetics of humoral response and its relationship with the disease severity in COVID-19
Source: Commun Biol. 2020 Dec 11;3:780. doi: 10.1038/s42003-020-01526-8 (PMC7733479; doi:10.1038/s42003-020-01526-8)
Supplement: Supplementary file 1 — Supplementary Information [file 42003_2020_1526_MOESM1_ESM.pdf]

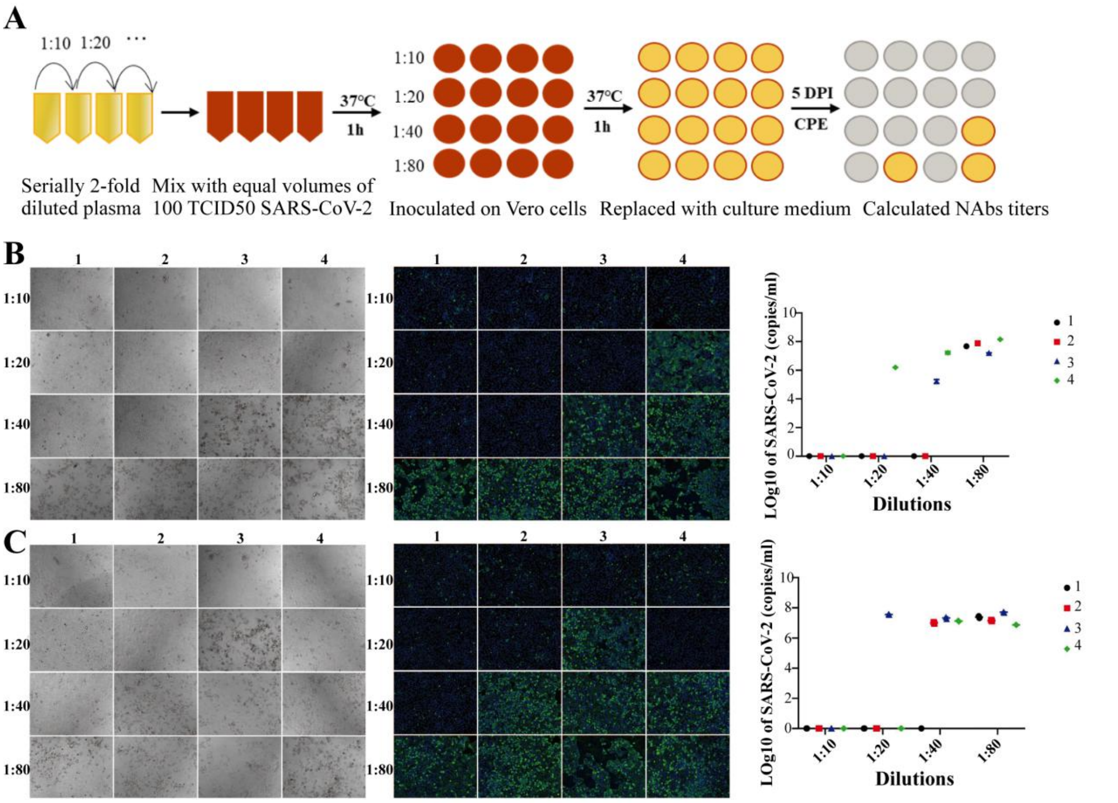

Supplementary Fig. 1 Design of microneutralization assays for the plasma. (A) The Schematic of the neutralization assay protocol. The plasma samples were diluted serially at two-fold (1:10 to 1:320), then mixed with equal volumes of SARS-CoV-2 at a dose of 100 TCID50 (50% tissue culture infective dose) at 37°C for 1h. Then 100μl mixture was added in quadruplicate on Vero cells cultured in 96-well microtiter plates at 37°C for 1h. The virus-plasma mixture was removed and replaced with 200ul fresh maintain medium. The neutralizing effects were determined at 5 days post viral infections according to the cytopathic effects. **(B)** & **(C)** Representative neutralizing results of two plasma samples determined by immunofluorescence assay and viral RNA quantification. The NAbs titers were 1:40 and 1:28.6, respectively. The cytopathic effects were observed by using Optical Microscope (10 × magnification). The viral replication in cells was assessed by immunofluorescence using an inhouse anti-nucleocapsid antibody (green). Nuclei were stained with DAPI (blue). The

nucleic acids were extracted from cultured medium in each serially diluted plasma treated well and viral RNA was analyzed by using qRT-PCR. The number indicated the well of the cell culture plate.

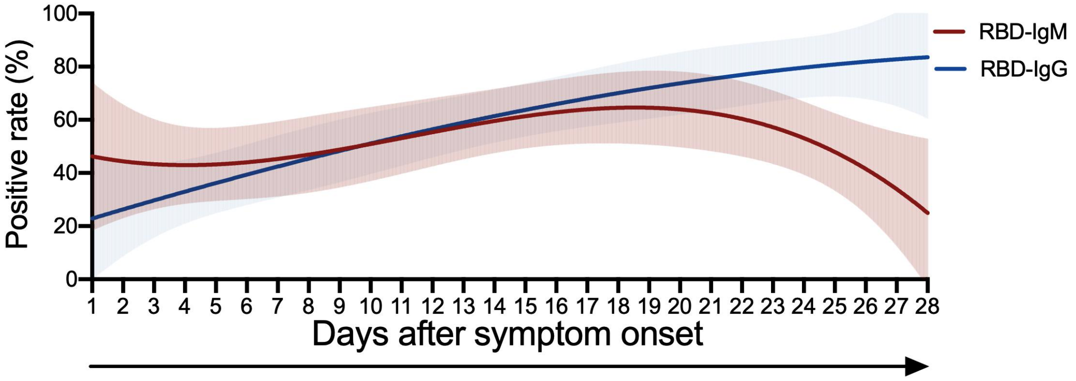

Sample numbers	1	2	3	4	5	6	7	8	9	10	11	12	13	14	15	16	17	18	19	20	21	22	23	24	25	26	27	28
RBD-IgM	1	5	3	4	4	3	6	8	8	5	8	15	6	8	12	11	4	6	9	1	2	8	5	1	3	1	1	0
RBD-IgG	1	3	1	4	1	4	5	7	5	7	8	15	7	11	15	12	4	6	7	5	3	8	5	3	3	1	2	1
Total	3	7	10	13	8	10	9	16	13	16	13	22	15	13	18	16	9	10	11	8	3	9	6	4	5	2	2	1

Supplementary Fig. 2 The positive rate of anti-receptor binding domain (RBD) IgM and IgG antibodies in all the tested plasma samples over time. The fitted cure lines are created by Fit Spline program of Graphpad software. The 95% confidence interval are shown for each curve. The lower table show the number of samples tested positive at each time point.

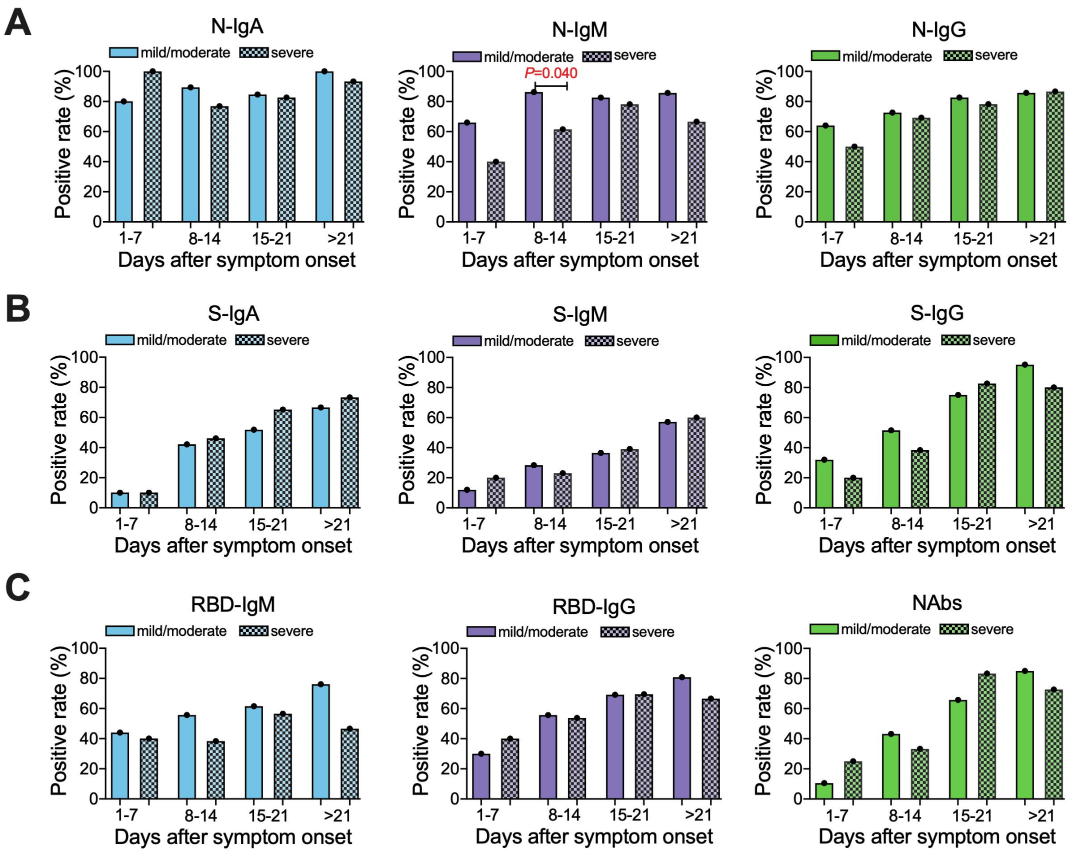

Supplementary Fig. 3 The weekly positive rates of antibodies against SARS-CoV-2 in patients with different severity. The positive rates of IgA, IgM and IgG antibodies against N, S and RBD proteins in plasma samples collected from mild/moderate and severe patients at different time points after symptom onset were calculated based on ELISA tests. The neutralizing antibodies (NAbs) were measured by microneutralization assay. The data of each group are shown weekly. Positive rates of IgA, IgM and IgG antibodies against N **(A)** and S **(B)**. Positive rates of IgM and IgG antibodies against RBD and of NAbs **(C)**.

Supplementary Table 1. The demographic information and clinical manifestations of recruited COVID-19 patients.

	Total (Cases=176)	Mild/moderate (Cases =140)	Severe (Cases=36)	*P* value*
Age, years				0.004
Median (IQR)	48 (40.0-57.8)	47 (39.0-56.0)	54 (42.5-66.8)	..
Gender, n (%)				0.261
Male	113 (64.2)	87 (62.1)	26 (72.2)	..
Female	63 (35.8)	53 (37.9)	10 (27.8)	..
Underlying diseases, n (%)				..
None	93 (52.8)	75 (53.6)	18 (50.0)	0.702
Yes	83 (47.1)	65 (46.4)	18 (50.0)	0.702
Hypertension	33 (18.8)	27 (19.3)	6 (16.7)	0.720
Surgery history	27 (15.3)	23 (16.4)	4 (11.1)	0.430
Diabetes	12 (6.8)	6 (4.3)	6 (16.7)	0.018
Coronary heart disease	7 (4.0)	6 (4.3)	1 (2.8)	1.000
Respiratory disease	10 (5.7)	7 (5.0)	3 (8.3)	0.429
Stroke	3 (1.7)	1 (0.7)	2 (5.6)	0.107
Other diseases	42 (23.9)	32 (22.9)	10 (27.8)	0.537
Symptoms, n (%)				..
Fever	151 (85.8)	117 (83.6)	34 (94.4)	0.096
Cough	136 (77.3)	106 (75.7)	30 (83.3)	0.331
Dyspnea	56 (31.8)	31 (22.1)	25 (69.4)	<0.001
Fatigue	44 (25.0)	29 (20.7)	15 (41.7)	0.010
Muscle pain	37 (21.0)	20 (14.3)	17 (47.2)	<0.001
Headache	31 (17.6)	17 (12.1)	14 (38.9)	<0.001
Sore throat	24 (13.6)	9 (6.4)	15 (41.7)	<0.001
Diarrhea	7 (4.0)	4 (2.9)	3 (8.3)	0.152

Clinical Outcome, n (%)				<0.001
Recovery	166 (94.3)	140 (100.0)	26 (72.2)	..
Death	10 (5.7)	0 (0.0)	10 (27.8)	..
ICU, n (%)				<0.001
Yes	15 (8.5)	1 (0.7)	14 (38.9)	..
No	161 (91.5)	139 (99.3)	22 (61.1)	..
Hospital stay, median (IQR)	19 (14.0-26.0)	18 (13.0-24.0)	27 (19.3-36.0)	<0.001

* *P* values were calculated by two-sided unpaired t test or χ^2 test as appropriate, where P<0.05 was considered to be statistically significant.

ICU, intensive care unit. IQR, interquartile range.

Supplementary Table 2. Laboratory findings of recruited COVID-19 patients.

	Total (Cases=176)	Mild/Moderate (Cases =140)	Severe (Cases=36)	*P* value*
Hemoglobin, g/L	128.6 (126.0-131.2)	129.7 (126.9-132.5)	124.2 (117.2-131.1)	0.093
Albumin, g/L	35.8 (34.8-36.7)	36.6 (35.6-37.6)	32.6 (30.7-34.5)	0.001
Globulin, g/L	30.3 (29.7-31.0)	29.9 (29.3-30.6)	31.9 (30.3-33.5)	0.013
Alanine aminotransferase, U/L	43.1 (37.3-48.9)	41.6 (35.8-47.4)	49.0 (31.3-66.7)	0.314
Total bilirubin, μmol/L	12.5 (11.3-13.6)	11.7 (10.6-12.9)	15.3 (12.0-18.7)	0.046
Creatinine, μmol/L	72.7 (69.0-76.4)	70.4 (67.4-73.3)	81.9 (67.7-96.0)	0.114
Procalcitonin, ng/mL	0.1 (0.1-0.2)	0.1 (0.1-0.1)	0.3 (0.0-0.7)	0.197
Erythrocyte, $\times 10^{12}$ per L	4.3 (4.2-4.4)	4.3 (4.2-4.4)	4.2 (3.9-4.4)	0.091
Leucocytes, $\times 10^{9}$ per L	6.6 (6.0-7.1)	5.9 (5.4-6.5)	9.0 (7.5-10.5)	<0.001
Platelets, $\times 10^{9}$ per L	220.9 (206.9-234.9)	217.3 (202.3-232.3)	234.8 (197.2-272.4)	0.322
Neutrophils, $\times 10^{9}$ per L	5.0 (4.4-5.5)	4.3 (3.8-4.8)	7.6 (6.2-9.1)	<0.001
Lymphocytes, $\times 10^{9}$ per L	1.1 (1.1-1.2)	1.2 (1.1-1.3)	0.9 (0.7-1.1)	0.009
Eosinophils, %	0.6 (0.5-0.8)	0.7 (0.5-0.9)	0.4 (0.1-0.7)	0.120
Basophils, %	0.2 (0.2-0.2)	0.2 (0.2-0.2)	0.2 (0.2-0.3)	0.386

* *P* values were calculated by two-sided unpaired t test, where $P<0.05$ was considered to be statistically significant.

Supplementary Table 3. Antibody positive rate and appearance time in plasma samples of the COVID-19 patients in the study.

	Total (Cases=176) (Samples=279)	Mild/modertate (Cases =140) (Samples=218)	Severe (Cases=36) (Samples=61)	*P* value*
Days from disease onset to antibody detection, median (IQR)	14.0 (7.0-18.8)	13.0 (6.3-17.0)	16.5 (8.0-22.8)	0.015
SARS-CoV-2 antibody, n (%)				
N-IgA positive	243 (87.1)	190 (87.2)	53 (86.9)	0.956
N-IgM positive	216 (77.4)	176 (80.7)	40 (65.6)	0.012
N-IgG positive	207 (74.2)	162 (74.3)	45 (73.8)	0.932
S-IgA positive	119 (42.7)	86 (39.4)	33 (54.1)	0.041
S-IgM positive	87 (31.2)	64 (29.4)	23 (37.7)	0.213
S-IgG positive	162 (58.1)	124 (56.9)	38 (62.3)	0.449
RBD-IgM positive	152 (54.5)	123 (56.4)	29 (47.5)	0.218
RBD-IgG positive	158 (56.6)	121 (55.5)	37 (60.7)	0.473
NAbs positive**	87 (49.4)	65 (46.4)	22 (61.1)	0.116
Antibody appearance time, median (IQR)				
N-IgA	13.0 (8.0-17.0)	12.0 (8.0-16.0)	15.0 (9.5-22.0)	0.005
N-IgM	13.0 (9.0-17.8)	12.0 (9.0-16.0)	16.5 (12.3-21.8)	<0.001
N-IgG	14.0 (10.0-18.0)	13.0 (9.0-16.0)	18.0 (12.5-22.5)	<0.001
S-IgA	15.0 (12.0-20.0)	14.0 (11.0-18.0)	19.0 (15.0-23.0)	0.008
S-IgM	15.0 (11.0-21.0)	14.0 (10.3-18.8)	19.0 (15.0-25.0)	0.009
S-IgG	15.0 (11.8-19.3)	14.0 (11.0-18.0)	18.5 (15.0-23.0)	0.001
RBD-IgM	13.5 (9.0-18.0)	12.0 (9.0-16.0)	17.0 (12.0-20.5)	0.037
RBD-IgG	14.5 (11.0-19.0)	14.0 (10.0-17.5)	17.0 (12.5-22.0)	0.046
NAbs	17.0 (13.0-22.0)	16.0 (12.0-19.0)	19.0 (15.0-23.0)	0.353

Antibody levels (OD value), mean (95% CI)				
N-IgA	0.29 (0.26-0.32)	0.29 (0.26-0.32)	0.29 (0.23-0.35)	0.997
N-IgM	0.27 (0.24-0.29)	0.27 (0.25-0.30)	0.24 (0.19-0.29)	0.229
N-IgG	0.69 (0.64-0.75)	0.68 (0.62-0.74)	0.74 (0.62-0.86)	0.394
S-IgA	0.17 (0.16-0.19)	0.16 (0.15-0.18)	0.22 (0.17-0.26)	0.023
S-IgM	0.18 (0.16-0.19)	0.18 (0.16-0.19)	0.17 (0.15-0.20)	0.949
S-IgG	0.28 (0.26-0.31)	0.28 (0.25-0.31)	0.29 (0.24-0.34)	0.651
RBD-IgM	0.26 (0.24-0.27)	0.26 (0.24-0.28)	0.23 (0.20-0.27)	0.220
RBD-IgG	0.47 (0.43-0.51)	0.45 (0.41-0.49)	0.57 (0.46-0.67)	0.033
NAbs[#]	1.13 (1.05-1.20)	1.11 (1.03-1.19)	1.20 (1.05-1.36)	0.282

* *P* values were calculated by two-sided unpaired t test or χ^2 test as appropriate, where $P<0.05$ was considered to be statistically significant.

**The neutralizing antibodies were analyzed based on only one sample of patients.

[#]The levels of neutralizing antibodies were expressed by end point titers.

IQR, interquartile range; Ig, immunoglobulin; N, nucleocapsid; S, spike; RBD, receptor binding domain; NAbs, neutralizing antibody. OD, optical density.
