# Supplementary material for: The kinetics of humoral response and its relationship with the disease severity in COVID-19
Source: Commun Biol. 2020 Dec 11;3:780. doi: 10.1038/s42003-020-01526-8 (PMC7733479; doi:10.1038/s42003-020-01526-8)
Supplement: Supplementary file 2 — Description of Additional Supplementary Files [file 42003_2020_1526_MOESM2_ESM.pdf]

Description of Additional Supplementary Files

File Name: Supplementary Data 1
Description: Supplementary Data 1 is about the basic information of patients/samples and antibody levels.

File Name: Supplementary Data 2
Description: Supplementary Data 2 is the source data underlying plots shown in figures.
