# Supplementary material for: The kinetics of humoral response and its relationship with the disease severity in COVID-19
Source: Commun Biol. 2020 Dec 11;3:780. doi: 10.1038/s42003-020-01526-8 (PMC7733479; doi:10.1038/s42003-020-01526-8)
Supplement: Supplementary file 5 — Reporting Summary [file 42003_2020_1526_MOESM5_ESM.pdf]

nature research

Corresponding author(s): Jianwei Wang, Dingyu Zhang, Linghang Wang and Lokesh Sharma

Last updated by author(s): Oct 15, 2020

Reporting Summary

Nature Research wishes to improve the reproducibility of the work that we publish. This form provides structure for consistency and transparency in reporting. For further information on Nature Research policies, see our Editorial Policies and the Editorial Policy Checklist.

Statistics

For all statistical analyses, confirm that the following items are present in the figure legend, table legend, main text, or Methods section.

n/a	Confirmed	
☐	☒	The exact sample size (*n*) for each experimental group/condition, given as a discrete number and unit of measurement
☐	☒	A statement on whether measurements were taken from distinct samples or whether the same sample was measured repeatedly
☐	☒	The statistical test(s) used AND whether they are one- or two-sided<br>*Only common tests should be described solely by name; describe more complex techniques in the Methods section.*
☒	☐	A description of all covariates tested
☒	☐	A description of any assumptions or corrections, such as tests of normality and adjustment for multiple comparisons
☐	☒	A full description of the statistical parameters including central tendency (e.g. means) or other basic estimates (e.g. regression coefficient) AND variation (e.g. standard deviation) or associated estimates of uncertainty (e.g. confidence intervals)
☐	☒	For null hypothesis testing, the test statistic (e.g. *F*, *t*, *r*) with confidence intervals, effect sizes, degrees of freedom and *P* value noted<br>*Give P values as exact values whenever suitable.*
☒	☐	For Bayesian analysis, information on the choice of priors and Markov chain Monte Carlo settings
☒	☐	For hierarchical and complex designs, identification of the appropriate level for tests and full reporting of outcomes
☐	☒	Estimates of effect sizes (e.g. Cohen's *d*, Pearson's *r*), indicating how they were calculated

Our web collection on statistics for biologists contains articles on many of the points above.

Software and code

Policy information about availability of computer code

Data collection	No external data collection is used.
Data analysis	All statistical analysis were conducted using SPSS version 19.0 and R version 3.6.1. Details of publicly available software used in the study are given in the "Methods". No other custom code or mathematical algorithm that is deemed central to the conclusions was used.

For manuscripts utilizing custom algorithms or software that are central to the research but not yet described in published literature, software must be made available to editors and reviewers. We strongly encourage code deposition in a community repository (e.g. GitHub). See the Nature Research guidelines for submitting code & software for further information.

Data

Policy information about availability of data

All manuscripts must include a data availability statement. This statement should provide the following information, where applicable:

- Accession codes, unique identifiers, or web links for publicly available datasets
- A list of figures that have associated raw data
- A description of any restrictions on data availability

The source data underlying the graphs and charts in the main and supplementary figures are available in Supplementary Data.

Field-specific reporting

Life sciences study design

All studies must disclose on these points even when the disclosure is negative.

Sample size	A total of 279 plasma samples were collected from 176 COVID-19 patients from three cohorts, including 218 samples from 140 mild/moderate patients and 61 samples from 36 severe patients.
Data exclusions	All tested data were involved in the analysis. No data was excluded from analysis.
Replication	All experimental findings could be reliably reproduced.
Randomization	Randomization is not relevant to this study because this is not a clinical trial.
Blinding	Blinding is not relevant to this study because this is not a clinical trial.

Reporting for specific materials, systems and methods

We require information from authors about some types of materials, experimental systems and methods used in many studies. Here, indicate whether each material, system or method listed is relevant to your study. If you are not sure if a list item applies to your research, read the appropriate section before selecting a response.

Materials & experimental systems

n/a	Involved in the study	
☐	☒	Antibodies
☐	☒	Eukaryotic cell lines
☒	☐	Palaeontology and archaeology
☒	☐	Animals and other organisms
☐	☒	Human research participants
☒	☐	Clinical data
☒	☐	Dual use research of concern

Methods

n/a	Involved in the study	
☒	☐	ChIP-seq
☒	☐	Flow cytometry
☒	☐	MRI-based neuroimaging

Antibodies

Antibodies used	The polyclonal anti-human IgA (α chain specific) antibody from rabbit (Cat. 309-035-011), anti-human IgM antibody (Fc5μ fragment specific) (Cat. 109-035-043) from goat were from Jack Immuno Research Inc (West Grove, PA, USA). The anti-human IgG (Fc specific) antibody produced in goat (Cat. A0170) was from Sigma-Aldrich (St. Louis, MO, USA).
Validation	The human plasma was used as primary antibody. The results were determined according to the cut-off value. Part of the IgG-positive results were validated by using western blotting.

Eukaryotic cell lines

Policy information about cell lines

Cell line source(s)	Vero cells were from ATCC, and the code number was CCL-81.
Authentication	The cell line was authenticated for research by ATCC.
Mycoplasma contamination	The cell lines have been tested routinely to confirm no mycoplasma contamination.
Commonly misidentified lines (See ICLAC register)	No commonly misidentified lines were used in this study.

Human research participants

Policy information about studies involving human research participants

Population characteristics	A total of 176 COVID-19 inpatients were recruited from three independent cohorts, in which 113 (64.2%) were males. The patients were aged from 18 to 82 years (mean of 49.0). Majority of the patients had clinical symptoms included fever (151, 85.8%), cough (136, 77.3%), and dyspnea (56, 31.8%) upon admissions . Comorbidities in form of underlying diseases were recorded in 83 (47.1%) patients, including hypertension, diabetes, chronic respiratory diseases, coronary heart disease, stroke and etc.
Recruitment	The patients with diagnosis of COVID-19 during the study period were recruited after they signed on the written informed consent or waived according to the emerging status.
Ethics oversight	This study was approved by the Ethical Review Board of Wuhan Jinyintan Hospital, Infectious Disease Hospital of Heilongjiang Province, and Institute of Pathogen Biology, Chinese Academy of Medical Sciences & Peking Union Medical College.

Note that full information on the approval of the study protocol must also be provided in the manuscript.
